# Pulmonary Sarcoidosis Mimicking Eosinophilic Bronchiolitis Diagnosed by *Cutibacterium Acnes* Positivity

**DOI:** 10.1002/rcr2.70736

**Published:** 2026-08-25

**Authors:** Keishi Sugino, Hirotaka Ono, Mikako Saito, Miho Kobayashi, Seiji Igarashi

**Affiliations:** ^1^ Department of Respiratory Medicine Tsuboi Hospital Koriyama Fukushima Japan; ^2^ Department of Diagnostic Pathology Tsuboi Hospital Koriyama Fukushima Japan

**Keywords:** bronchiolitis, *Cutibacterium acnes*, eosinophilia, sarcoidosis, surgical lung biopsy

## Abstract

A 63‐year‐old woman with bronchial asthma presented with diffuse centrilobular nodules and bronchial wall thickening, initially suggesting eosinophilic bronchiolitis. Surgical lung biopsy revealed well‐formed non‐caseating granulomas with concomitant eosinophilic bronchiolitis, while *Cutibacterium acnes* immunostaining established a diagnosis of pulmonary sarcoidosis. This case highlights that pulmonary sarcoidosis can mimic eosinophilic bronchiolitis.

## Graphical abstract

Pulmonary sarcoidosis may closely mimic eosinophilic bronchiolitis in patients with established bronchial asthma. Surgical lung biopsy revealed well‐formed non‐caseating granulomas with concomitant eosinophilic bronchiolitis, and *Cutibacterium acnes* immunostaining supported the diagnosis of pulmonary sarcoidosis. Histopathological examination is essential for distinguishing these entities.

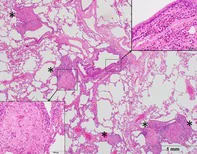

A 63‐year‐old woman with established bronchial asthma presented with progressive dyspnea, productive cough and weight loss. Despite maintenance inhaled corticosteroid/long‐acting β2‐agonist therapy for 3 years, her respiratory symptoms gradually worsened. Peripheral blood eosinophilia (19.6%) and elevated fractional exhaled nitric oxide (111 ppb) suggested active eosinophilic airway inflammation. Pulmonary function testing showed obstructive impairment with reduced maximal mid‐expiratory flow (0.37 L/s) and maximal expiratory flow at 25% of vital capacity corrected for height (0.11 L/s/m), suggesting peripheral airway involvement. High‐resolution computed tomography showed diffuse centrilobular nodules and bronchial wall thickening (Figure 1A,B). Bronchoalveolar lavage fluid demonstrated eosinophilia (21.6%) and lymphocytosis (28.2%), with a CD4/CD8 ratio of 1.7.

**FIGURE 1:**
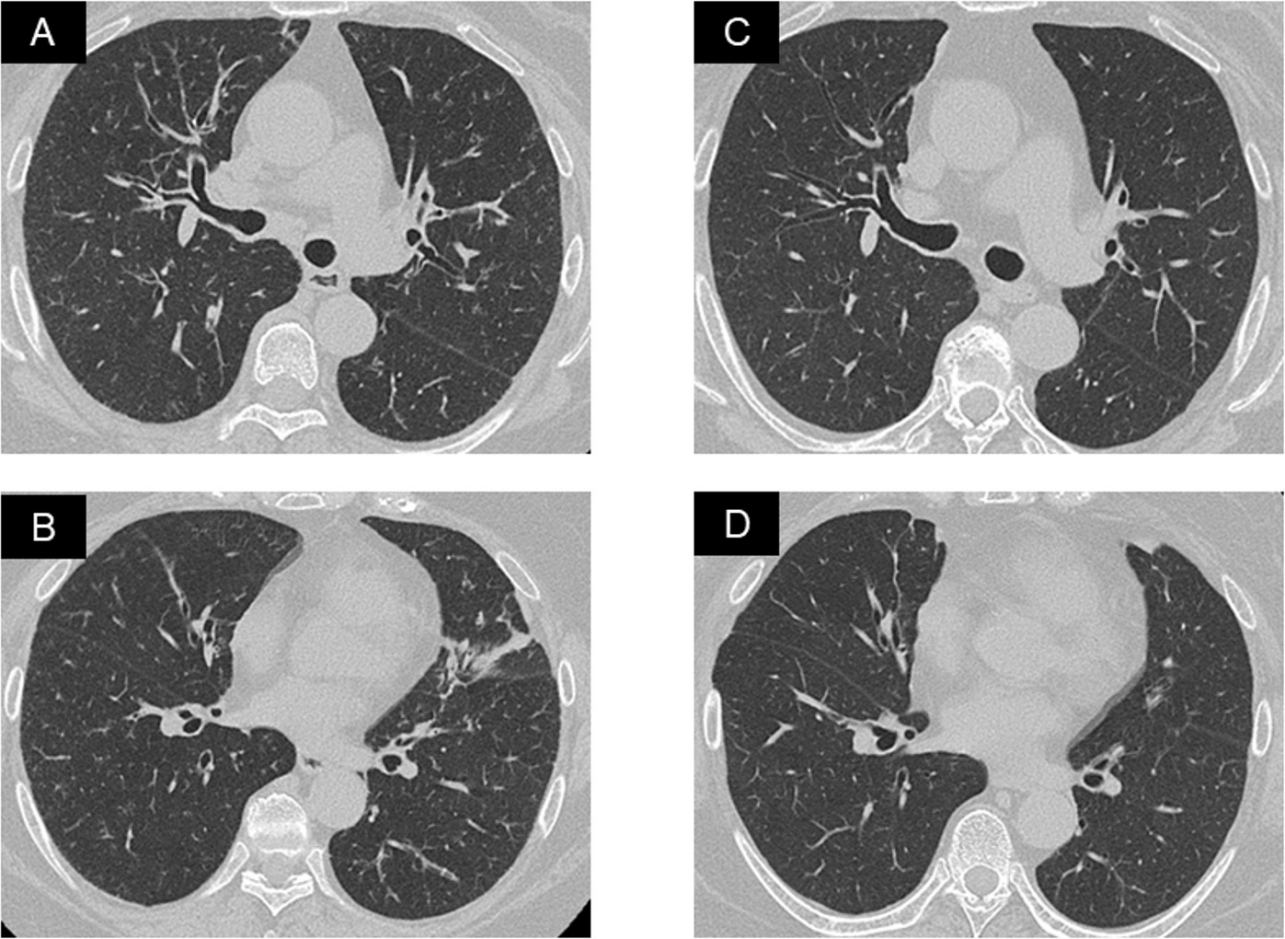
Chest computed tomography before and after corticosteroid therapy. (A, B) Pretreatment chest computed tomography demonstrating diffuse bilateral centrilobular nodules and bronchial wall thickening. (C, D) Follow‐up computed tomography after corticosteroid therapy showing marked improvement of the centrilobular nodules and airway lesions.

Histopathology revealed numerous well‐formed non‐caseating epithelioid granulomas distributed predominantly along the bronchovascular bundles. Representative granulomas contained multinucleated giant cells, and marked eosinophilic bronchiolitis with mucus plugging was also identified (Figure 2). Immunohistochemical staining demonstrated *Cutibacterium acnes* positivity within macrophages adjacent to granulomas (Figure 3), supporting the diagnosis of pulmonary sarcoidosis [1].

**FIGURE 2:**
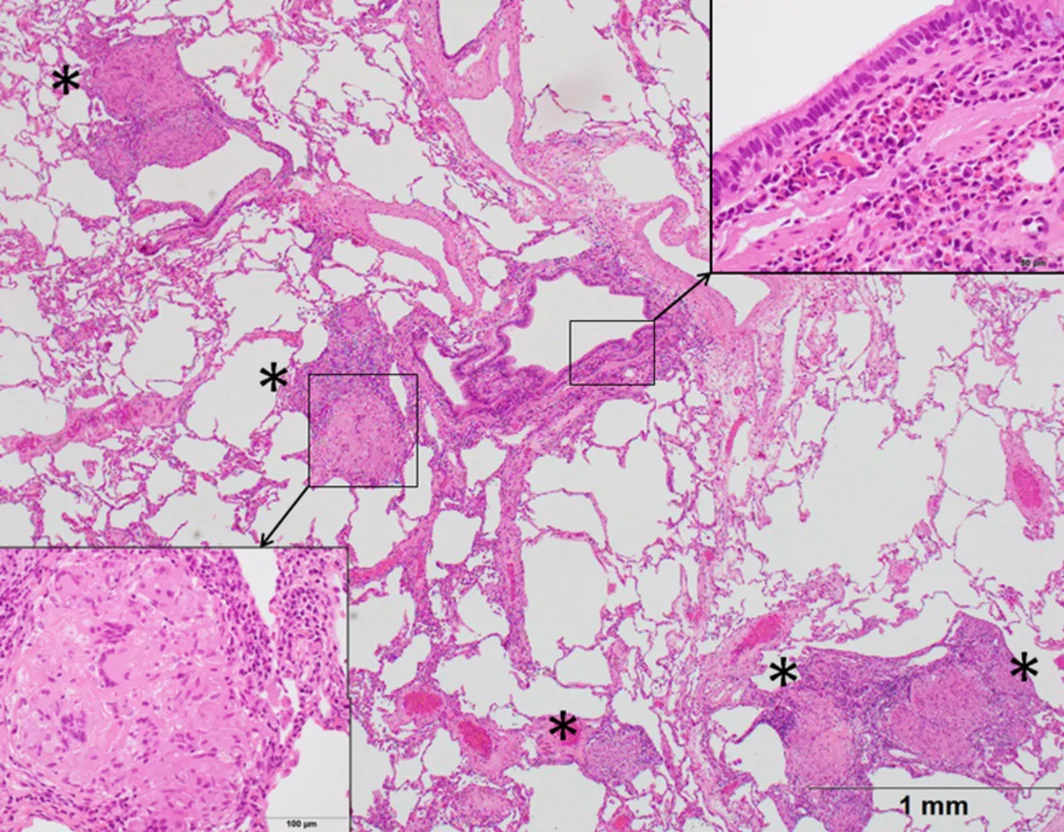
Histopathological findings of the surgical lung biopsy. Low‐power view showing multiple well‐formed non‐caseating epithelioid granulomas distributed predominantly along the bronchovascular bundles (asterisks). The upper right inset shows marked eosinophilic infiltration of the bronchiolar wall, consistent with eosinophilic bronchiolitis. The lower left inset demonstrates a higher magnification of a representative non‐caseating granuloma containing multinucleated giant cells. Scale bar = 1 mm in the main panel and 100 μm in the lower inset (haematoxylin and eosin stain).

**FIGURE 3:**
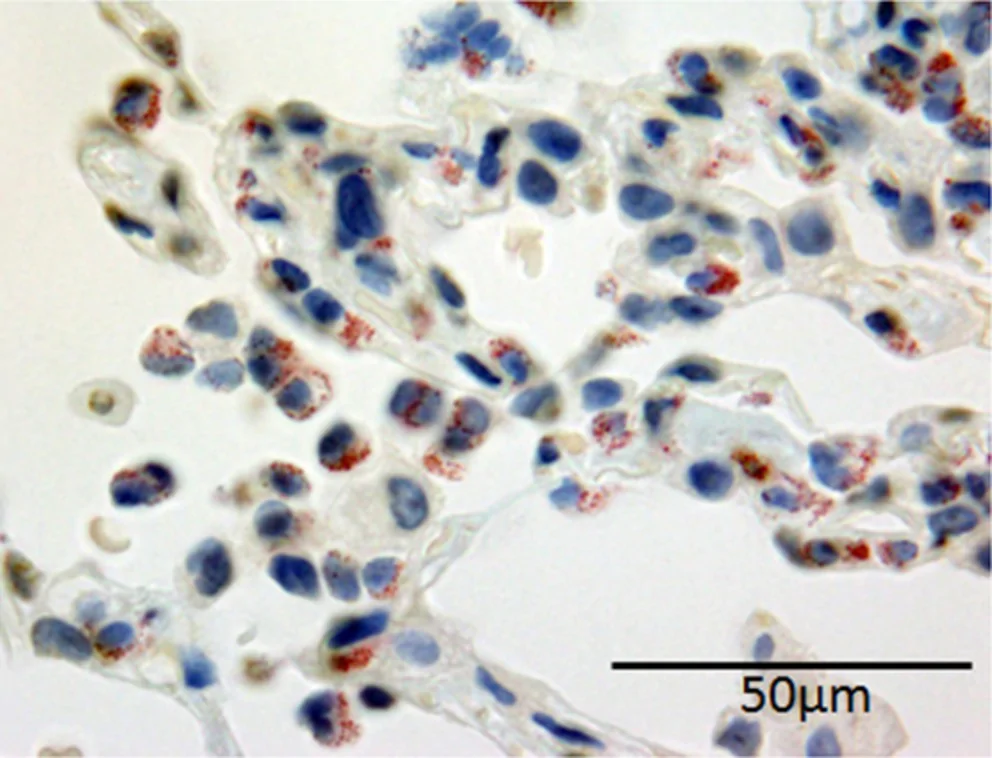
Immunohistochemical staining for *Cutibacterium acnes*. Immunohistochemistry using a monoclonal antibody against *Cutibacterium acnes* (PAB antibody) demonstrates positive staining within macrophages adjacent to granulomatous lesions, supporting the diagnosis of sarcoidosis. Scale bar = 50 μm.

The major differential diagnosis was eosinophilic bronchiolitis because of the patient's history of bronchial asthma, peripheral and bronchoalveolar eosinophilia and diffuse centrilobular nodules on high‐resolution computed tomography [2]. However, the presence of well‐formed non‐caseating epithelioid granulomas together with *Cutibacterium acnes* positivity established the diagnosis of pulmonary sarcoidosis. Corticosteroid therapy resulted in marked clinical and radiological improvement. No apparent trigger was identified, and the pathophysiological relationship between asthma and pulmonary sarcoidosis remains unclear.

This case highlights that pulmonary sarcoidosis may mimic eosinophilic bronchiolitis, even in patients with established bronchial asthma. Histopathological examination with *Cutibacterium acnes* immunostaining is essential for accurate diagnosis.

## Author Contributions

K.S. was responsible for conceptualization and drafting the manuscript. K.S., H.O. and M.S. were involved in the clinical management of the patient and data collection. M.K. and S.I. contributed to the pathological evaluation and interpretation of the findings. All authors contributed to data interpretation, critically revised the manuscript and approved the final version.

## Funding

The authors have nothing to report.

## Disclosure

The authors did not use any artificial intelligence (AI)–assisted technologies in the preparation of this manuscript.

## Consent

The authors declare that written informed consent was obtained for the publication of this manuscript and accompanying images using the consent form provided by the Journal.

## Conflicts of Interest

The authors declare no conflicts of interest.

## Data Availability

The data that support the findings of this study are available on request from the corresponding author. The data are not publicly available due to privacy or ethical restrictions.
